# Predictive Framework Based on GBIF and WorldClim Data for Identifying Drought- and Cold-Tolerant *Magnolia* Species in China

**DOI:** 10.3390/plants14131966

**Published:** 2025-06-27

**Authors:** Minxin Gou, Jie Xu, Haoxiang Zhu, Qianwen Liao, Haiyang Wang, Xinyao Zhou, Qiongshuang Guo

**Affiliations:** 1 School of Horticulture and Landscape Architecture, Southwest University, Chongqing 400715, China; messiah15683211236@163.com (M.G.); zhuhx8910@swu.edu.cn (H.Z.); 18075923727@163.com (Q.L.); whyswau@126.com (H.W.); 2 School of Resources and Environment, Southwest University, Chongqing 400715, China; 3 College of Landscape Architecture, Sichuan Agricultural University, Chengdu 611130, China; 15736051381@163.com; 4 Key Laboratory of Monitoring, Evaluation and Early Warning of Territorial Spatial Planning, Chongqing Institute of Planning and Design, Ministry of Natural Resources, Chongqing 400715, China; guoqiongshuang@163.com

**Keywords:** *Magnolia*, plant specimens, climate data, drought resistance, cold tolerance

## Abstract

This study developed a preliminary screening framework for identifying candidate *Magnolia* species potentially resistant to drought and cold conditions, using open access plant specimens and climate data. Based on 969 specimens, a distribution database was constructed to map 35 *Magnolia* species in China. Nonparametric variance analysis revealed significant interspecific differences in precipitation of the driest quarter (PDQ) and minimum temperature of the coldest month (MTCM). Using the updated climatic thresholds, nine candidate species each were identified as having drought resistance (PDQ < 60.5 mm) and cold tolerance (MTCM < 0.925 °C). In conclusion, the proposed method integrates geocoded specimen information with climate data, providing preliminary candidate species for future physiological validation, conservation planning, and further botanical research. However, the primary focus on climate data and lack of consideration of non-climatic factors warrant cautious interpretation of the results and comprehensive investigations for validation of the present study results.

## 1. Introduction

In plants, physiological, biochemical, and molecular processes are adversely affected by abiotic stress [1]. To combat abiotic stresses induced by climate change, most wild provenances have developed physiological and ecological strategies [2], eventually acquiring traits that are valuable for genetic breeding and agricultural production [3,4]. Morphological or physiological indicators have been extensively used to guide the selection of potentially resistant provenances [5,6,7]. However, a limited range of provenances have been studied to date because of biased scientific interest and the time-consuming nature of field collection, among other factors [8]. With the advancement of digital technology, the use of big data has emerged as an effective method for predicting plants’ abiotic stress tolerance, facilitating the identification of potential seed sources for testing [9,10,11]. Climate is a crucial factor affecting species distribution on a macro scale [12,13]. According to the prevailing scientific hypothesis, a plant’s tolerance to abiotic stressors is determined by the environmental conditions of its geographical distribution region. This concept has been widely applied in various fields, such as biodiversity protection [14], ecology [15], and evolutionary biology [16]. Studies have developed various methods to assess the resistance of herbaceous plants to abiotic stresses based on online plant and environmental datasets. However, these methods have not yet been widely applied to woody plants [17].

The Global Biodiversity Information Facility (GBIF, www.gbif.org) is the largest biological data portal that contains records for approximately 850 million species [18]. WorldClim (https://worldclim.org/) is a global dataset of spatially interpolated monthly climate data [19]. Simple species distribution models (SDMs), which are constructed by combining geographic information from extensive species data with environmental datasets, can be used to screen for drought- and extreme temperature-tolerant plant species [20,21,22].

*Magnolia* L. plants have variegated flowers, with a fragrant scent, and thrive in a warm and humid climate. Specifically, these plants are distributed in the warm temperate and subtropical climate zones, located in south of the Yellow River basin in China, with a gradually decreasing distribution trend toward the northeast and northwest regions [23,24]. Drought and low-temperature stress are key limiting factors for the growth and geographical distribution of *Magnolia* species, accounting for their limited utilization in northern city landscapes [25,26]. When introduced into alpine arid regions, *Magnolia* plants exhibit limited growth due to severe abiotic stresses. Thus, screening for drought and cold resistance characteristics of wild provenances is essential to cultivate resistant varieties [27]. China encompasses a range of climatic zones, including tropical, subtropical, warm temperate, and sub-cold regions. The origin of *Magnolia*, China boasts great diversity of both native and introduced varieties, with approximately 50 *Magnolia* species reported to date [28,29]. Substantial climate variations across China offer diverse growth environments for *Magnolia* species, making it an ideal setting for studying the resistance of *Magnolia* to drought and other abiotic stresses.

In the present study, we linked the geographic distribution data of online plant specimens with their climatic data available in WorldClim to determine the water and temperature conditions for each specimen. Based on these conditions, we screened each *Magnolia* species for potential tolerance to drought and cold. From a pool of 35 *Magnolia* species, we identified nine candidates each with drought and cold resistance. We used a comprehensive list supported by large-scale data records to ensure a wide coverage and prevent the oversight of less common species. Finally, we constructed a preliminary screening framework for assessing abiotic stress tolerance in *Magnolia* at the interspecific level.

## 2. Materials and Methods

### 2.1. Species Distribution Data

The distribution data of *Magnolia* specimens were retrieved from the GBIF, using “*Magnolia* Linn.” as the keyword and “China” as the filter by region. The data are accessible via GBIF.org at https://doi.org/10.15468/dl.ajyf67 (accessed on 13 October 2022) (GBIF Occurrence Download). Using ArcGIS 10.2 (Environmental Systems Research Institute, https://developers.arcgis.com/), we extracted GPS data from the downloaded *Magnolia* specimen records to create spatial coordinate points. WGS 84 was employed as the geographical coordinate system. To address potential systematic errors arising from uneven sampling intensity during the data analysis, we resampled the coordinate points within a 0.1° grid per species, randomly retaining only one coordinate point per grid. This resampling ensured the inclusion of *Magnolia* species with ≥10 specimens for further analysis. Moran’s I value was computed at 10, 20, and 50 km lag distances for each of the 35 species by using the “ape” package in R 4.3.3, which yielded coefficients indistinguishable from zero (I ≈ 0, *p* > 0.05). The results indicated spatial randomness in the records and a lack of any spatial sampling bias (Supplementary Table S1).

### 2.2. Climatic Data

Considering the extreme conditions that can limit species distribution and drawing on the analytical methods used in high-quality studies in this field, we considered the precipitation of the driest quarter (PDQ) and the minimum temperature of the coldest month (MTCM) as the predictive indices of resistance [20,21]. Climate data, including precipitation and temperature, were downloaded from WorldClim version 2.1 (https://www.worldclim.org/, accessed on 18 June 2025) at a 30-arc-second grid resolution (approximately 1 km^2^). These data were used to quantify the current climate scenario, focusing on the lowest temperatures and precipitation levels during the dry season.

### 2.3. Data Analysis

The map of China (reference number: GS (2019) 1669) was obtained from the “Data Service” section of the official website of the Ministry of Natural Resources (https://www.mnr.gov.cn/). By integrating this map with the coordinate points extracted from the GBIF, we obtained a distribution map of *Magnolia* species in China. Using the Spatial Analyst toolbox in GIS, climate data values from WorldClim were linked to these distribution points, and the results were tabulated in the. csv format. These tables were further processed in Microsoft Excel 2016 (Microsoft, Redmond, WA, USA, https://www.microsoft.com/zh-cn/). All data were analyzed in IBM SPSS 20.0 (International Business Machines Corporation, Armonk, NY, USA, https://www.ibm.com/analytics/spss-statistics-software, accessed on 20 October 2022) to create scatter plots and box plots depicting the relationship between *Magnolia* distribution and climatic conditions. For an enhanced visual representation, the diagrams were recolored in Adobe Photoshop CS 5.0 (Adobe Systems Incorporated, San Jose, CA, USA, https://www.adobe.com/cn/products/photoshop.html, accessed on 22 October 2022).

Interspecific variations within the *Magnolia* genus were examined using the Shapiro–Wilk test and nonparametric analysis of variance. Using the square-based Euclidean distance, a hierarchical clustering analysis was performed to group *Magnolia* species based on similar water and temperature conditions, specifically in terms of the median PDQ and MTCM. Scatter plots were generated to visually depict the relationship between each group of *Magnolia* and its corresponding water–heat conditions.

The median values of PDQ and MTCM were calculated as the indicators of *Magnolia* species’ tolerance to water and heat conditions, respectively. These values corresponded to the effect of water and temperature conditions on the distribution trend of *Magnolia* species. Guided by the hierarchical clustering analysis and the 25th percentiles of PDQ and MTCM across all species, the resistance thresholds of 60.5 mm and 0.925 °C were established for PDQ and MTCM, respectively. To validate this approach and assess the robustness of species classification, sensitivity analysis was conducted using 5th, 10th, 15th, 20th, 30th, and 35th percentiles. The results underscored the 25th percentile as the optimal threshold for eliminating type I and type II errors in species classification: using more stringent thresholds (5–10th percentiles) and inclusive thresholds (30–35th percentiles) presented the risks of excluding potentially tolerant species (type II error) and including marginally tolerant species (type I error), respectively. This approach aligns with established practices in ecological threshold analysis where quartile-based boundaries effectively balance sensitivity and specificity in species classification (Supplementary Table S2). Based on these thresholds, we classified the specimens as candidate drought-tolerant and cold-tolerant species. To assess whether differences in the environmental conditions between the tolerant and susceptible specimens were significant, their spatial distributions along PDQ and MTCM gradients were compared using the Mann–Whitney U test, with the wilcox.test() function in R 4.3.3. Additionally, Rolland’s method was used to calculate the niche width for the resistant taxa, defined as the difference between the maximum and minimum values of the environmental variables for each species [30].

## 3. Results and Analysis

### 3.1. Distribution Status of Magnolia in China

Specimen records of 73 datasets were downloaded from the GBIF. Following resampling within a 0.1° grid, the GPS data from these specimens were linked with the climate data, and PDQ and MTCM values were extracted to the respective points. Specimens with missing climate data were excluded. Finally, 969 specimens were retained to construct a comprehensive distribution database, comprising geographic coordinates, PDQ, and MTCM of the 35 *Magnolia* species. Spatial autocorrelation analysis confirmed the lack of significant spatial bias among the included specimens (Moran’s I ≈ 0, *p* > 0.05; Supplementary Table S1), indicating that the constructed dataset was free from sampling artifacts commonly associated with GBIF occurrence records.

**Figure 1:**
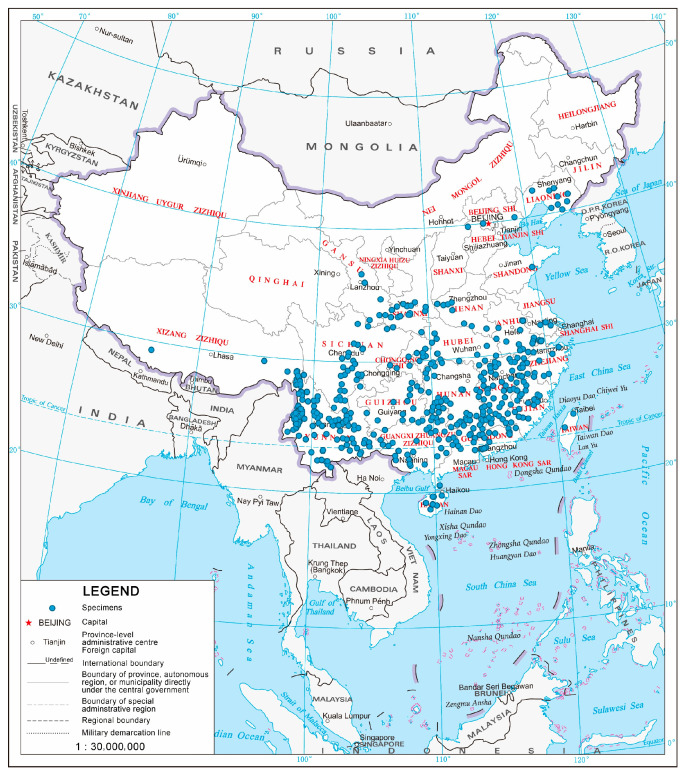
Distribution of *Magnolia* L. in China.

Based on the geographic coordinates for the 969 specimens, derived from ArcGIS, we created a map of *Magnolia* species distribution in China (Figure 1). The results indicated that *Magnolia* species are primarily distributed in the Guangdong, Guangxi, Yunnan, and Hunan provinces, along with widespread distribution in Zhejiang, Hubei, Jiangxi, and Sichuan provinces, and scattered occurrences in Northern and Northeastern China.

### 3.2. Significant Interspecific Variations in Magnolia Distribution Induced by Climate Change

**Figure 2:**
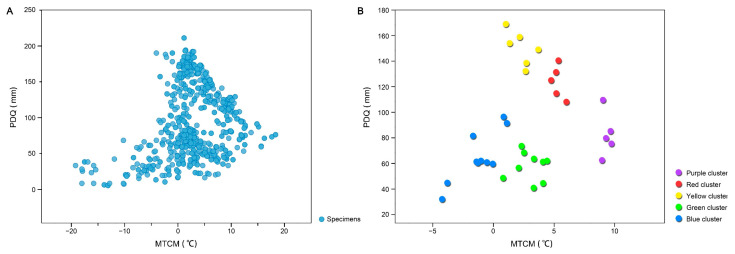
The climate conditions of *Magnolia*. (**A**) The distribution of *Magnolia* specimens in their corresponding climate conditions. (**B**) The median values of the precipitation of the driest quarter (PDQ) and the minimum temperature of the coldest month (MTCM) for 35 *Magnolia* species. The species were divided into five clusters by using the hierarchical clustering method based on square Euclidean distance.

The normality of PDQ and MTCM values for the genus *Magnolia* was assessed using the Shapiro–Wilk test. A *p* value of <0.05 indicated that the null hypothesis (H0) was invalid, confirming that the data did not follow a normal distribution. Thus, a nonparametric analysis of variance based on the Kruskal–Wallis test was conducted to analyze interspecific variations. The results indicated that the interspecific differences in PDQ and MTCM across *Magnolia* genus in China were significant (*p* < 0.05; Table 1). To illustrate the water–heat tradeoff for *Magnolia* species under natural conditions, a simple SDM was constructed (Figure 2A). The linear goodness of fit, indicated by an R^2^ value of <0.5, confirmed a poor model fit. This finding, in conjunction with the Kruskal–Wallis test results, reinforced the presence of significant interspecific variations within the *Magnolia* genus.

Using median climatic conditions for hierarchical clustering analysis, 35 *Magnolia* species were stratified into five clusters (Figure 2B). These clusters differed in their PDQ and MTCM values. The blue cluster, with species like *M. biondii* and *M. campbellii*, had the lowest MTCM (−5 °C to 2 °C) and moderate PDQ (30–100 mm), indicating tolerance to both temperature and moisture variations. The purple cluster, including *M. alba* and *M. champaca*, showed high MTCM (8–11 °C) and moderate PDQ (60–120 mm), reflecting a need for more temperature and moisture. The green cluster, with species such as *M. albosericea* and *M. wilsonii*, had concentrated MTCM values (0–5 °C) and moderate PDQ (40–80 mm), suggesting resilience to low-temperature conditions. The yellow cluster had the highest PDQ (120–170 mm) and moderate MTCM (0–4 °C), indicating adaptation to higher precipitation and milder temperatures. The red cluster showed intermediate values of both MTCM (4–6 °C) and PDQ (100–140 mm) (Table 2).

To validate the robustness of threshold selection for the subsequent classification of tolerant species, sensitivity analysis was conducted across multiple percentile thresholds (5th to 35th). The analysis confirmed that the 25th percentile threshold optimally balanced classification stringency with inclusiveness: six species each were consistently classified as drought-tolerant and cold-tolerant species (appearing in ≥5 of 7 threshold scenarios), with *M. biondii* and *M. globosa* exhibiting the greatest consistency across all threshold levels (Supplementary Table S2). This statistical validation reinforced the reliability of species classification for subsequent analyses.

### 3.3. Magnolia Species with Potential Drought Resistance

**Figure 3:**
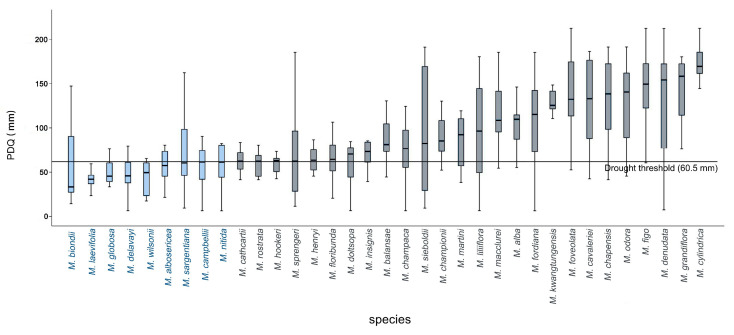
Drought-tolerance potential of *Magnolia* species. Blue boxes represent candidate drought-tolerant species, and gray boxes represent nontolerant species.

Using the PDQ data from 969 *Magnolia* specimens, climatic conditions of the native distribution ranges of *Magnolia* species were evaluated. The corresponding boxplot is shown in Figure 3 and Supplementary Table S3. Significant interspecific differences were observed in PDQ values. Based on these results, combined with those of hierarchical clustering analysis, we screened nine *Magnolia* species as candidates for potential drought resistance. A PDQ threshold of <60.5 mm was used to screen for drought-resistant species. The species were ranked from the strongest to the weakest in terms of potential drought resistance as follows: *M. biondii*, *M. laevifolia*, *M. globosa*, *M. delavayi*, *M. wilsonii*, *M. albosericea*, *M. sargentiana*, *M. campbellii*, and *M. nitida.*

**Figure 4:**
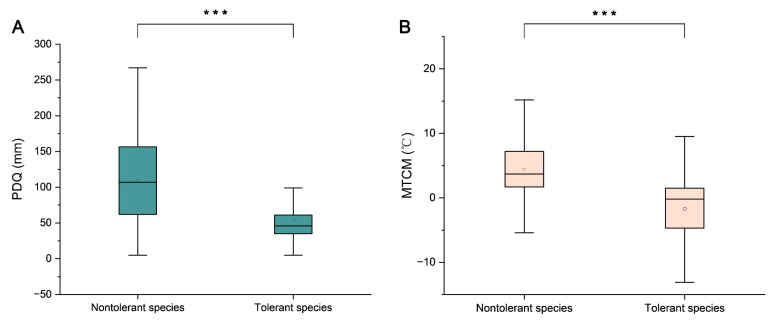
Climatic niche differentiation between the tolerant and nontolerant species. (**A**) Precipitation of the driest quarter (PDQ) for candidate drought-tolerant vs. nontolerant species. (**B**) Minimum temperature of the coldest month (MTCM) for candidate cold-tolerant vs. nontolerant species. *** indicates *p* < 0.001 (Mann–Whitney U test).

The Mann–Whitney U test revealed that the environmental differences between drought-tolerant (PDQ < 60.5 mm) and nontolerant species (PDQ ≥ 60.5 mm) were significant (*p* < 0.01) (Figure 4A). Among the candidate drought-tolerant species, *M. biondii* exhibited the greatest adaptability and the broadest niche width, whereas *M. laevifolia*, *M. globosa*, and *M. delavayi* demonstrated limited adaptability to varying water conditions.

### 3.4. Magnolia Species with Potential Cold Resistance

The MTCM values for the native environments of *Magnolia* species ranged from −4 °C to 13 °C. As illustrated in Figure 4 (also detailed in Supplementary Table S3), the sampled specimens demonstrated significant interspecific differences in the temperatures experienced during the coldest season. Based on the findings of previous studies on low-temperature freezing damage in *Magnolia* plants [31,32], and considering the 25th percentile of MTCM values across all species, an MTCM of <0.925 °C was established as the threshold for cold tolerance. Using this criterion, nine *Magnolia* species were identified as candidate cold-tolerant species. These species ranked from the strongest to the weakest in terms of the potential cold tolerance as follows: *M. biondii*, *M. globosa*, *M. sieboldii*, *M. rostrata*, *M. campbellii*, *M. sprengeri*, *M. nitida*, *M. sargentiana*, and *M. liliiflora*.

**Figure 5:**
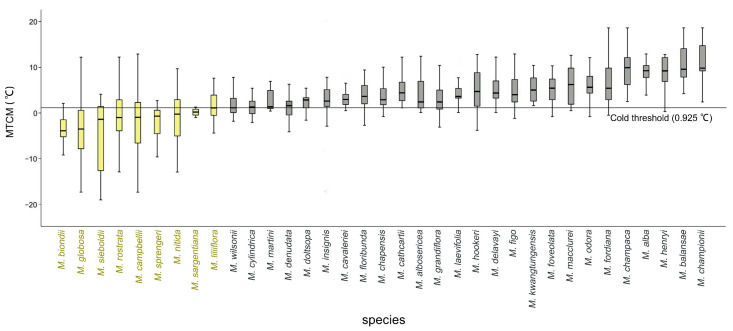
Cold-tolerance potential of *Magnolia* species. Yellow boxes represent candidate cold-tolerant species, and gray boxes represent nontolerant species.

The classification of candidate cold-tolerant species was further validated using the Mann–Whitney U test. The environmental differences between the species predicted as cold-tolerant (MTCM < 0.925 °C) and nontolerant (MTCM ≥ 0.925 °C) were found to be significant (*p* < 0.01) (Figure 4B). Among the nine *Magnolia* species, *M. sieboldii* exhibited the maximum cold tolerance and widest niche width, whereas *M. zeii* and *M. amoena* displayed relatively small niche widths and weak resistance (Figure 5).

## 4. Discussion

In China, *Magnolia* species have long been cultivated and hold significant ecological and economic value. However, most species of this genus are sensitive to low-temperature and drought conditions [33]. Interestingly, natural provenances have preserved a high level of genetic diversity through centuries of evolutionary selection, providing a crucial material basis for developing resistant or high-quality breeds [34,35]. Many scholars have investigated the response of *Magnolia* species to abiotic stresses by using physiological and molecular tests, offering vital theoretical support for their introduction, domestication, and genetic breeding [31,36,37]. Despite these advances, collecting wild resources poses practical challenges, and establishing a comprehensive evaluation system for the abiotic stress resistance of *Magnolia* species in China remains a time-intensive and challenging task. Climate is a primary factor affecting the spatial and temporal distributions of plants on a large scale [38]. Applying this concept, scholars have employed reverse modeling to predict species adaptation to environmental changes based on shifts in their ecological niches. This approach has also facilitated matching of the ecological types of urban forestry trees and the prediction of thermotolerant and cold-tolerant horticultural crops [8,17]. Building on this, the present study used the digital knowledge-sharing platform GBIF to acquire GPS data for *Magnolia* specimens in China. Integrating GPS data with WorldClim climate data, we developed a method for evaluating potential drought and cold tolerance within *Magnolia* genus.

The proposed method is based on the actual ecological niche of *Magnolia* species and considers factors such as interspecies interaction and diffusion ability. However, modeling the actual ecological niche is associated with an inherent analytical bias [39], which may lead to an underestimation of environmental tolerance. Despite this, modeling species distribution provides a useful screening approach. Existing research, through large-scale data analysis, has provided a theoretical foundation for exploring abiotic stress tolerance and climate conditions [40,41]. In summary, this method enables the rapid and straightforward screening of potentially tolerant provenances before laboratory testing. Specifically, it establishes a preliminary species list that can be directly subjected to physiological validation, thereby reducing efforts at introduction and domestication.

Through sensitivity analyses, this study selected the 25th percentile threshold for species classification, at which an optimal balance was achieved between classification stringency and biological inclusiveness (Supplementary Table S2). More restrictive thresholds (5–10th percentiles) identified only 2–4 species, potentially excluding marginally tolerant candidates that could be valuable for conservation or breeding programs. Conversely, more inclusive thresholds (30–35th percentiles) captured 9–12 species, indicating an overestimation of species’ tolerance capabilities. The 25th percentile threshold revealed a core group with stable trends, with six species each classified consistently as candidate drought-tolerant and cold-tolerant species across multiple threshold scenarios; *M. biondii* and *M. globosa* showed the highest consistency (appearing in all seven threshold levels). Although no single threshold can be universally considered “valid,” this analysis demonstrates that threshold levels can be optimized through a comparative analysis of classification stability. Furthermore, the 25th percentile threshold serves as a conservative proxy that approximates the lower climatic limits of species’ realized niches, where physiological stress responses are likely to intensify. This approach aligns with ecological modeling practices and is supported by global patterns of plant stress tolerance observed in field studies of woody species under extreme environmental conditions [40,41].

Based on the set thresholds for drought and cold tolerance, that is, PDQ < 60.5 mm and MTCM < 0.925 °C, respectively, our preliminary screening framework revealed 18 drought- and cold-tolerant provenances of *Magnolia*. Among these species, *M. biondii* and *M. globosa* showed both drought and cold resistance. Some of the candidate species screened in this study have already been reported in the literature. For example, the predicted drought-resistant species, namely *M. laevifolia*, is endemic to Yunnan, with natural distribution in regions with moderately dry conditions, indicating that it has strong ecological adaptability to arid environments [42]. *M. delavayi*, which often grows in arid mountain environments, is an endangered plant endemic to southwest China and holds great economic value in urban landscapes for its lotus-like milky-white flowers [43]. In terms of cold tolerance, *M. sieboldii* thrives in cool and humid climate and is the only wild *Magnolia* plant naturally distributed in northeast China [44]. *M. biondii* and *M. sprengeri* are mostly distributed in north of the Yangtze River [45]. Their habitat suitability, assessed using Maxent model, indicates not only their strong cold tolerance but also the tendency to inhabit high latitudes characterized by low temperatures and less precipitation [46].

Several *Magnolia* species identified in this study as having potential drought and cold tolerance are notably endangered or rare, which poses a seemingly paradoxical issue (Supplementary Table S4) [47,48]. For instance, *M. globosa* was predicted to have strong tolerance to both drought and cold stresses, yet it is listed in the IUCN Red List, predominantly due to severe habitat fragmentation and loss resulting from human disturbances, rather than inherent biological vulnerabilities [49]. Similarly, *M. rostrata* and *M. zenii*, predicted as cold-tolerant species, are naturally confined to specific ecological niches [49,50]. *M. zenii* has a highly localized distribution and exists in fragmented populations that make it vulnerable to extinction despite its physiological resilience. *M. wilsonii*, predicted as a drought-resistant species, typically occupies specialized ecological habitats, which are often at risk due to human activities, rather than from abiotic factors alone [48]. This paradox—stress tolerance coupled with endangered status—can be explained in the ecological and anthropogenic contexts. While these *Magnolia* species exhibit intrinsic ability to tolerate drought or cold, their rarity stems primarily from habitat loss and human disturbances, rather than physiological limitations. Additionally, China’s current climate warming and moistening trend may diminish their adaptive advantages, further threatening these specialized habitats. Therefore, conservation and practical utilization of these stress-tolerant species depend on habitat protection, restoration, and ex situ preservation. Of note, species predictions based on relatively few specimens (fewer than 15 occurrences, e.g., *M. globosa* and *M. rostrata*) inherently carry a high risk of uncertainty, warranting cautious interpretation and further validation of the data.

The findings of this study offer theoretical insights that can aid in the preservation of *Magnolia* diversity, introducing new perspectives for future breeding endeavors aimed at producing resistant varieties. These findings have several practical applications across different domains. First, through a screening of potentially tolerant species with enhanced survival potential under future climate scenarios, this study offers valuable resources to inform ex situ conservation priorities. Second, in urban landscaping, the predicted tolerant species represent valuable candidates for greening initiatives in regions with intensifying drought or cold stress conditions. Third, for plant breeding applications, the identified species constitute valuable genetic resources for developing climate-resilient cultivars through targeted hybridization or selective breeding approaches, with a particular emphasis on incorporating stress tolerance traits into commercially viable horticultural varieties.

Some limitations of our approach warrant careful consideration. First, our prediction method relies solely on species presence data, without explicitly incorporating absence data or differentiating between potential causes of the observed distribution patterns. Species distributions are shaped not only by physiological climatic tolerance but also significantly by historical biogeographical processes (e.g., past climatic refugia and historical dispersal routes), dispersal constraints that weaken species’ colonization ability, and biotic interactions including interspecific competition or facilitation [51]. For example, habitat fragmentation and human-driven land-use changes could isolate populations, severely constraining dispersal and artificially narrowing species distribution ranges independent of their intrinsic physiological tolerances. Consequently, the observed distributions may reflect historical contingencies and anthropogenic disturbances, rather than purely climatic factors, thus potentially confounding predictions of true physiological stress tolerance.

Second, although our correlative analysis establishes statistical associations between *Magnolia* species distributions and selected climate variables, it could not verify causal relationships among these variables. In addition, this study did not consider abiotic factors, other than PDQ and MTCM, which also hold ecological relevance and can influence drought tolerance, such as potential evapotranspiration and soil moisture dynamics. Nevertheless, previous urban forest studies have validated PDQ as an effective drought stress indicator, revealing its strong correlations with the resistance potential of trees under extreme conditions [52,53].

Finally, the conservation status and narrow distribution ranges of several species predicted as being stress tolerant (e.g., *M. globosa* and *M. zenii*) emphasize a crucial distinction between climatic suitability inferred from distribution data and actual physiological tolerance. Rare species restricted to fragmented habitats might be physiologically resilient yet remain vulnerable due to non-climatic pressures. Thus, our findings represent general hypotheses on *Magnolia* species stress tolerance, requiring cautious interpretation and rigorous experimental validation under controlled physiological experiments [54].

Future studies should incorporate more sophisticated species distribution modeling approaches, such as ensemble modeling techniques that combine multiple algorithms, or mechanistic models that explicitly account for physiological processes, to provide a nuanced understanding of the relationships between species distributions and environmental factors [55]. Additionally, integrating available data for species absence, along with those for biotic factors, would provide a more comprehensive understanding of the factors limiting species distribution [56]. Experimental validation to bridge the critical gap between the correlative distribution patterns and actual physiological tolerance capabilities also holds significance [57]. To validate the drought tolerance of the candidate species, future studies should conduct water stress experiments under controlled conditions involving graduated soil water content treatments, focusing on the measurements of leaf water potential, osmolyte accumulation (proline, soluble sugars), antioxidant enzyme activities (SOD, POD, CAT), and electrolyte leakage rates [58]. For cold-tolerance assessment, graduated temperature stress trials are warranted to evaluate membrane stability, focusing on electrolyte leakage, chlorophyll fluorescence parameters (Fv/Fm ratios), and visual damage scoring [59]. To provide definitive validation of our distribution-based preliminary screening approach, we recommend future comparative trials between the species predicted as tolerant and nontolerant under standardized stress conditions and using regression models to quantify the relationships between distribution-derived safety margins and measured physiological tolerance indices [60]. Such validation experiments would establish whether climatic niche parameters effectively predict actual physiological capabilities, thereby strengthening the scientific foundation for climate-based species selection in conservation and horticultural applications.

## 5. Conclusions

Building on the well-established biogeographical theory regarding the significant influence of climate on large-scale species distribution, we propose a method to predict the abiotic stress tolerance of Magnolia plants. Leveraging online specimen data, we effectively utilized a vast number of plant samples to comprehensively analyze Magnolia resources in China. Eighteen candidate species requiring experimental validation for abiotic stress resistance were identified. The proposed approach can enhance practitioners’ ability to gather information and prevent oversight of certain populations. The proposed framework can be useful in effective resource identification at the interspecies level and in the rapid screening of tolerant provenances, facilitating targeted conservation planning and breeding. However, predictions based solely on climate data must be interpreted cautiously, as species distributions may also be influenced by non-climatic factors such as habitat fragmentation, dispersal limitations, interspecies competition, historical events, and inherent uncertainties from limited specimen records. For scientific validation of the data predicted in the present study, we recommend follow-up controlled physiological experiments, involving drought gradient trials assessing leaf water potential and osmotic adjustments and cold stress tests measuring chlorophyll fluorescence. Finally, application of the proposed methodology to additional plant genera is essential to strengthen its reliability and broaden its applicability.

## Figures and Tables

**Table 1:** Kruskal–Wallis test of corresponding environmental conditions in *Magnolia*.

Environmental Condition	Sample Size	Testing Statistic	Degree of Freedom	*p* Value of Two-Sided Test
PDQ	969	391.68	34	0.00
MTCM		411.39		

**Table 2:** Grouping of *Magnolia* under water–heat tradeoff relationship.

Clusters	*Magnolia* Taxa	PDQ/mm	MTCM/°C
Purple cluster	*M. alba* (16), *M. balansae* (16), *M. champaca* (18), *M. championii* (15), *M. henryi* (17)	60~120	8~11
Red cluster	*M. fordiana* (57), *M. foveolata* (41), *M. kwangtungensis* (12), *M. macclurei* (25), *M. odora* (28)	100~140	4~6
Yellow cluster	*M. cavaleriei* (24), *M. chapensis* (22), *M. cylindrica* (20), *M. denudata* (77), *M. figo* (114), *M. grandiflora* (19)	120~170	0~4
Green cluster	*M. albosericea* (14), *M. cathcartii* (24), *M. delavayi* (32), *M. doltsopa* (26), *M. floribunda* (43), *M. hookeri* (14), *M. insignis* (51), *M. laevifolia* (24), *M. wilsonii* (18)	40~80	0~5
Blue cluster	*M. biondii* (21), *M. campbellii* (24), *M. globosa* (11), *M. liliiflora* (41), *M. martini* (12), *M. nitida* (14), *M. rostrata* (11), *M. sargentiana* (11), *M. sieboldii* (26), *M. sprengeri* (41)	30~100	−5~2

## Data Availability

The original contributions presented in the study are included in the article/Supplementary Materials; further inquiries can be directed to the corresponding authors.

## Supplementary Materials

The following supporting information can be downloaded at: https://www.mdpi.com/article/10.3390/plants14131966/s1. Supplementary Table S1. Spatial autocorrelation (Moran’s I) analysis for 35 Magnolia species; Supplementary Table S2. Sensitivity analysis of percentile-based thresholds and corresponding candidate Magnolia species with drought and cold tolerance; Supplementary Table S3. Summary of the specimen distribution and climate metrics (precipitation of the driest quarter and minimum temperature of the coldest month) for 35 Magnolia species; Supplementary Table S4. IUCN Red List categories and endemism status for four Magnolia species identified as stress-tolerant candidates.
